# NF-κB pathway activators as potential ageing biomarkers: targets for new therapeutic strategies

**DOI:** 10.1186/1742-4933-10-24

**Published:** 2013-06-20

**Authors:** Carmela R Balistreri, Giuseppina Candore, Giulia Accardi, Giuseppina Colonna-Romano, Domenico Lio

**Affiliations:** 1 Department of Pathobiology and Medical and Forensic Biotechnologies, University of Palermo, Corso Tukory 211, Palermo 90134, Italy

**Keywords:** Biological ageing process, Inflammatory network and its effects in ageing, NF-κB signaling pathway as hub of inflammatory ageing network, Inflammatory biomarkers

## Abstract

Chronic inflammation is a major biological mechanism underpinning biological ageing process and age-related diseases. Inflammation is also the key response of host defense against pathogens and tissue injury. Current opinion sustains that during evolution the host defense and ageing process have become linked together. Thus, the large array of defense factors and mechanisms linked to the NF-κB system seem to be involved in ageing process. This concept leads us in proposing inductors of NF-κB signaling pathway as potential ageing biomarkers. On the other hand, ageing biomarkers, represented by biological indicators and selected through apposite criteria, should help to characterize biological age and, since age is a major risk factor in many degenerative diseases, could be subsequently used to identify individuals at high risk of developing age-associated diseases or disabilities. In this report, some inflammatory biomarkers will be discussed for a better understanding of the concept of biological ageing, providing ideas on eventual working hypothesis about potential targets for the development of new therapeutic strategies and improving, as consequence, the quality of life of elderly population.

## Introduction

**Figure 1:**
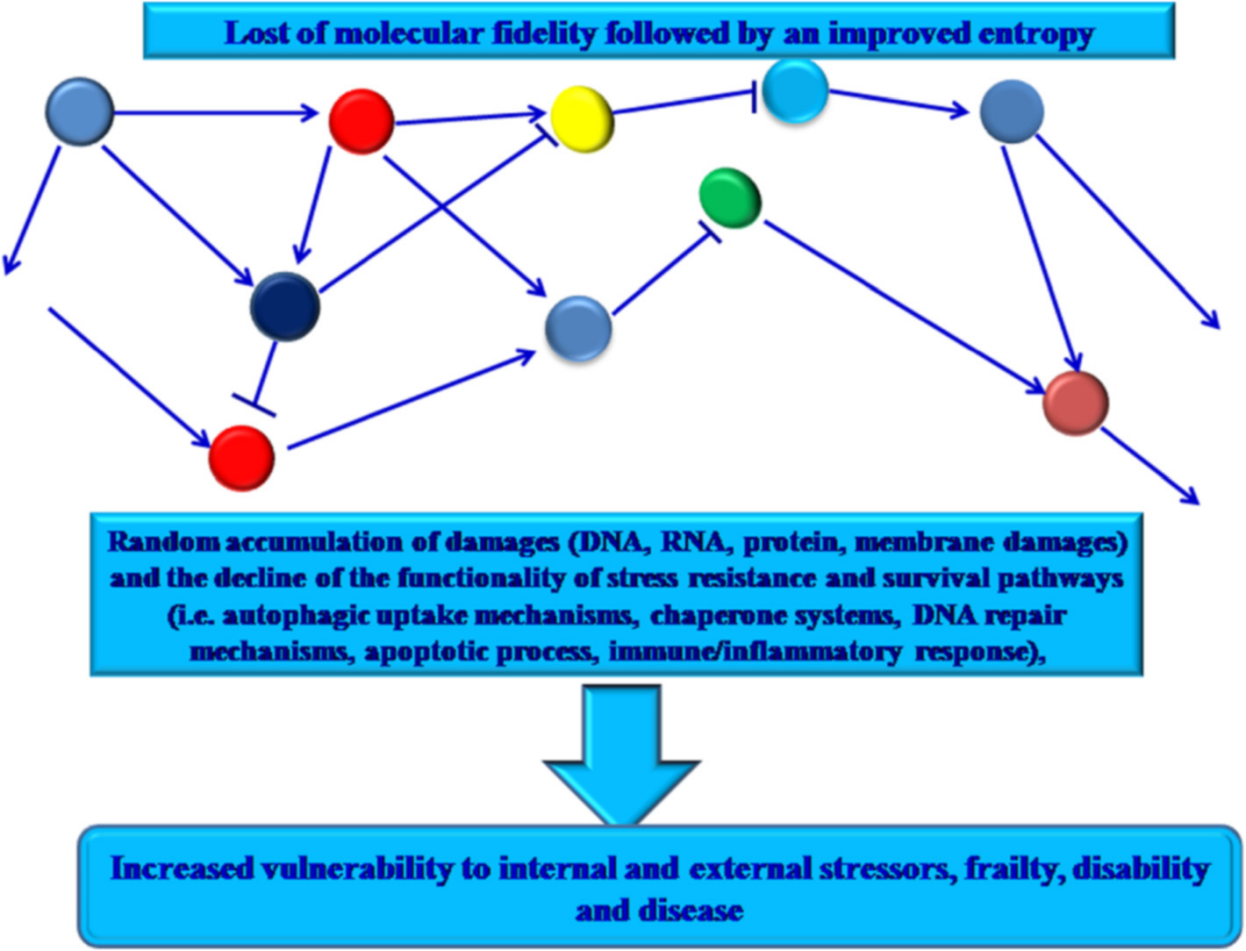
**Ageing process results by the lost of molecular fidelity followed by an improved entropy.** This determines loss complexity and random accumulation of damages (i.e. particularly damages to nuclear and mitochondrial DNA) at cellular, tissue, organ levels and/or of whole body. Thus, it becomes more easily vulnerable to internal and external stressors, frailty, disability and disease.

Ageing is a complex process, induced by an intricate interaction of genetic, epigenetic, stochastic and environmental factors. They determine the lost of molecular fidelity followed by an improved entropy [1,2]. As result, loss complexity and random accumulation of damages (i.e. particularly damages to nuclear and mitochondrial DNA) at cellular, tissue, organ levels and/or of whole body arise, compatibly with the disposable soma theory of ageing [3]. Thus, it establishes a condition, which modifies both architecture and functioning of physiological processes and regulatory (*immune and endocrine*) systems. This determines a deterioration of the homeostasis. Accordingly, it becomes more easily vulnerable to internal and external stressors, frailty, disability and disease (Figure 1). On the other hand, the loss of DNA integrity, the principal random damages able in modifying cellular fidelity and inducing cellular and whole body senescence, determines the decline of the functionality of stress resistance and survival pathways (i.e. autophagic uptake mechanisms, chaperone systems, DNA repair mechanisms, apoptotic process, immune/inflammatory response), involved in cellular and organism defense to environmental stress and maintaining homeostasis [2]. However, a large heterogeneity in occurrence, complications, speed, and age and gender manifestation of ageing process at cellular, tissue, organ levels and/or of whole body has been observed in humans. Among human people, there are individuals at the age ≥90 years still in good mental and physical conditions, and others that at the age ≥ 60 years show cognitive difficulties, and/or the onset of chronic inflammatory diseases, such as Alzheimer’s disease (AD), cardiovascular disease (CVD) and type 2 diabetes mellitus (T2DM) and cancer [4].

The principal causes of the heterogeneity in human ageing rate, measured as decline of functional capacity and stress resistance, seem to be genetic and environmental factors. However, the overall impression is that environmental factors are the major determinants of both ageing and age-related diseases [4,5]. Thus, ageing process is not a genetically programmed process [5]. This consideration is based on studies on heritability of age-related diseases and ageing [6,7]. A similar value of heritability in lifespan and age-related diseases, such as cancer, AD, CVD and T2DM, has been identified (35% *vs*. 40%, respectively) [8,9]. However, this does not imply that genetic factors have an irrelevant role in ageing and age-related diseases. For example, mutations identified in familial forms of AD consented understanding its molecular mechanisms, such as the toxicity of amyloid β peptide and potential therapeutic targets in more common sporadic late onset AD [10]. Common suggestion is based on both a complex contribution of genetic factors in ageing and diseases of later life and weak effects of individual genes [5]. Furthermore, diverse genetic factors are associated with ageing and exceptional longevity. Human genome-wide genetic analyses have revealed only few age-related loci and polymorphic longevity genes [11-13]. Among these, current promising candidates are Sirtuins, Forkhead box O protein (FoxOs) and the field of epigenetics. Functional genomics, i.e. expression profiling studies, have revealed a group of genes which are differently expressed in ageing, such as immune/inflammatory genes [14].

From the observations described above, another critical point of ageing process emerges based on the concept of biological age as real expression in human of both ageing rate and onset of the common diseases of later life rather than chronological age [15]. This concept opened an important area of research focused on identifying of potential molecular targets as *biomarkers of human biological ageing*[16]. On the one hand, it could consent to develop potential anti-ageing treatment strategies. On the other hand, probable anti-ageing treatments could retard or prevent age-associated diseases resulting in widespread health, social and economic benefit. Such treatment could include genetic engineering, such as gene therapy or endogenous gene repair, or pharmacological therapies, or changes in lifestyle, i.e. physical activity, diet.

In this report, many of these aspects are discussed, giving particular emphasis in describing some biomarkers of inflammation. In particular, the data discussed in this report are based on an expert opinion derived on the findings from author’s studies on ageing, age-related diseases and inflammation.

### Definition and selection criteria of ageing biomarkers

As established by National Institute of Health, a biomarker is a “*feature objectively measured and evaluated as an indicator of normal biologic processes, pathogenic processes, or pharmacologic responses to a therapeutic intervention”*[16,17].

In the case of ageing process, this definition might concern measures related to physical changes, such as grey hairs, reduced skin elasticity, wrinkles, reduced muscle strength or changes in the near vision, which are thought to be the result of molecular mechanisms occurring in the old age [18]. However, they reflect the chronological age rather than the biological age, which is the most important indicator of health and potential lifespan [15].

A biomarker of real ageing should reflect a process of biological ageing, be easily reproducible in cross-species comparison, be easily obtainable. Since ageing is the result of the deterioration of more than one system or process, it would be more appropriate to consider different biomarkers. Panels of biomarkers associated with conditions, alterations or changes of a set of critical systems to assess the biological age of any organism should be used [16,18].

The gerontologists have begun to face this problem already in the early 1980s, with the development of a large number of ageing biomarkers [16,18]. Despite the numerous efforts and the support in this research from National Institute of Ageing, the major number of biomarkers is still to date under discussion, like inflammatory markers, hormones, markers of oxidative stress or telomere shortening [16,18]. Most (perhaps all) markers are also not really proven in longitudinal studies in humans. In addition, they have been considered for a variety of purposes, which are not distinguished sufficiently. Most studies used biomarkers as tools for comparing ageing rate in several populations or cohorts of a single population. In contrast, others considered biomarkers for person-specific predisposition, which represents much more challenging, principally because ageing, as a biological process, is not well defined at individual level. In addition, the research of comparative or predictive biomarkers have determined the attempted use of measure panels associated with survival, health of old age, frailty, age-related (multi) morbidity and mortality [16,18].

However, none of the identified biomarkers is a “*real*” biomarkers of ageing. They are commonly related to age and diseases. In addition, the major number has been developed and tested for diseases in which biological age is the single biggest risk factor, such as peripheral blood cellular telomere length, indicators of immuno-senescence, even without correlations with disease-specific diagnoses. In addition, biomarkers of age-related diseases and ageing have been preferentially identified in younger-old populations (typically aged 60–85), but not in oldest-old (aged 85 and above) [16,18]. For example, blood pressure, indicators of metabolic syndrome and telomere length do not associate significantly with age-related morbidity or mortality in population-based studies of the oldest-old [19-21]. Thus, biomarkers of ageing and age-related diseases in understanding the health trajectories of the oldest-old are unexplored. It is important that this lacuna is filled given the rapid growth in the number of very old people in many contemporary populations.

For an ageing biomarker, it is important to know not only its definition, but also the criteria for its selection. Accordingly, the American Federation for Ageing Research suggested detailed criteria, recently reviewed by Sprott [16] and Johnson [18]. Based on these criteria, a true biomarker of ageing, in order to be both accurate and useful, should predict a person’s physiological, cognitive and physical function in an age-related way. In the same time, it should be easily testable, and not harmful to test individuals. For example, it could be a blood test or an image technique, by performing accurately and reproducibly without the need for specialized equipment or techniques. It should be tested preliminary in laboratory animals, such as mice, and successively in humans. Thus, a biomarker needs to be simple and inexpensive to use. They should cause little or no pain and stress (see Table 1) [16,18].

**Table 1:** Criteria for a biomarker of ageing process

	
**I**	It must predict the rate of ageing. In other words, it would estimate where a person is in their total lifespan. Operationally, it must be a better predictor of lifespan than chronological age alone.
**II**	It must monitor a basic process that underlines the ageing process, not the effects of diseases.
**III**	It must be able to be tested repeatedly without harming the person, for example, a blood test or an imaging techniques.
**IV**	It must be something that works in humans and in laboratory animals, such as mice. This is so that it can be tested in laboratory animals before being validated in humans.

Furthermore, current research on ageing biomarkers is also focusing in identifying molecules which also are able in giving clinical indications. On the other hand, biomarkers represent a hot topic and have the ability to change our life, if real prediction, on an individual basis, can be made in the future.

### Description and ageing biological effects of inflammatory network

Immune system is evolved to defend the host against microbial invasion, and to counteract tissue damage elicited by chemical or physical agents or trauma, maintaining consequently the homeostasis and tissue repair [22]. In both conditions, it responds in a appropriate manner by inducing apposite reactions (i.e. of suitable degree, with the involvement of a different array of cells and mediators), called *inflammatory responses*[22,23]. Inflammatory responses can be evocated initially as localized tissue reactions and subsequently as *acute phase reaction*, represented by systemic cytokine-induced reactions, including leukocytosis, fever, somnolence, anorexia, activation of hypothalamic-pituitary-adrenal axis and increased level of glucocorticoids, and acute phase synthesis, i.e. C reactive protein (CRP), in the liver. A complex network of molecules (the *mediators*) and cells (*neutrophils, monocytes, mast cells, endothelial cells, etc.*) characterize these reactions. They work together in concert and interact mediating the activation of different signaling pathways and the expression and transcriptional regulation of hub genes. The hub genes receive and direct the activity of many other genes [22,23]. Thus, these responses are induced through an inflammatory network. Recent studies on topology of this network evidence the crucial role of some mediators in driving the different cellular interactions and regulating the type of inflammatory reaction. Several mediators as pro- and anti-inflammatory molecules are involved [24]. Their release is modulated by different factors linked to well-known Nuclear Factor (NF)-κB pathway [25,26]. In addition, the magnitude of their production varies individually because of genetic heterogeneity. Single nucleotide polymorphisms (SNPs) in several genes and epigenetic factors seem to be involved [27]. Among the inflammatory mediators, the classical pro-inflammatory cytokines, Tumor necrosis factor α (TNF-α), Interleukin-1 (IL-1) and IL-6, play a key role. They are able in inducing both local and systemic effects [28]. When the causes of the inflammatory reaction are of a high intensity, their production is increased. Thus, they are released in the circulation provoking the acute phase response. In contrast, the anti-inflammatory cytokines, such as IL-10 are able to regulate the activation of inflammatory cells, by inhibiting the release of pro-inflammatory cytokines and therefore turning off the inflammatory processes [29].

Whether tissue health is not restored or in response to stable low grade irritation, inflammation becomes a chronic condition provoking continuous damages in the surrounding tissues. The collateral damage caused by this type of inflammation usually accumulates slowly, sometimes asymptomatically for years but can eventually lead to severe tissue deterioration [30].

From the above, it emerges that the inflammatory response is not *per se* a negative phenomenon. It is programmed by the evolution in neutralizing infectious agents, playing a beneficial role until the time of reproduction and parental care. In contrast, in old age, in a period largely not foreseen by evolution, it can determine a detrimental effect through chronic inflammatory responses (“antagonistic pleiotropy”) in several/ all tissue and organs, which are cause of both the ageing phenotype and chronic diseases [24,30]. A low chronic grade of inflammation, the *“inflammageing”*, characterized by a 2 to 4-fold increase in serum levels of inflammatory mediators has been identified in ageing [31]. It seems to be as optimal predictor of mortality and, as mentioned above, a critical risk factor in the pathogenesis of several age-related chronic diseases as AD, CVD, T2DM, sarcopenia, frailty and functional disability [32].

Augment of age-related body fat and consequent increase of visceral adiposity, age-related decline of sex hormones, oxidative and genotoxic stress, cellular and tissue damage, nutrition, alterations of physical condition of gut microbiota, other organs (brain, liver) and systems (immune and endocrine) have been associated with inflamm-ageing [32-34]. In addition, factors linking to physiological stress, such a long-term smoking and depression, seem also to contribute to inflammageing [32-34]. However, the most important factor for age-related inflammation is the long-life pathogen burden [30]. Some recent studies have, indeed, evidenced associations between past infections and levels of chronic inflammation and increased risk of heart attack, stroke, and cancer [32,34]. For instance, persistent peripheral multibacteria infection, such as periodontitis, associated with gram-negative anaerobic bacteria capable of exhibiting localized and systemic infections in the host, is considered as possible aggravating cofactor in subjects with vascular diseases and risk factor for the onset of other age-related diseases, such as AD [30].

Of special relevance is the inflammation status in centenarian people. The literature data seem to be apparently contradictory. Increased levels of both inflammatory and anti-inflammatory mediators and significant frequencies of protective genotypes have been assessed in centenarians than the old subjects [30,35]. As consequence, identifying of apposite biomarkers likely in long lived subjects should be necessary. This might permit a preferential and selected development of pleiotropic therapeutic interventions acting concomitantly on different targets and at different levels.

### Inflammatory ageing biomarkers: the crucial role of NF-κB activators

Ageing is not a genetically programmed process, as described above [5]. In contrast, it is recognized as an entropic process, characterized by loss of molecular fidelity and subsequent accumulation of different products [1,2]. In addition, it has been recently proposed that during evolution the host defense and the ageing process have become linked together [2]. Host defense and ageing mechanisms seem to be overlapping. In particular, host defenses seem to be involved in ageing process, to active inflammatory network and also to evocate the release of so-called *senescence associated secretory phenotype* (SASP), represented by a myriad of factors, such as the pro-inflammatory mediators [36,37]. A large range of defense factors and mechanisms are involved in inducing of inflammatory network, and are all (or the major number) linked to the NF-κB pathway, an ancient signaling pathway specialized to the host defense [25,26]. In particular, the NF-κB system is a cytoplasmatic sensor constituted by a protein-complex (Rel family proteins-RelA/p65, c-Rel and RelB- and NF-κB components-p50/p105 and p52/p100) and inhibited commonly by binding to IκB proteins (IκBα, IκBβ, IκBγ, IκBδ, IκBϵ, IκBζ and Bcl3). In some cases, its inhibition is induced through the action of several signaling pathways and negative feedback loops acting through different mechanisms at various levels of signaling cascades. In contrast, its activation can be evocated both by immune insults and external and internal danger signals associated with senescence and ageing process, such as oxidative and genotoxic stress and tissue injuries [25]. Namely, its induction is linked to several recognition pathway, i.e. Toll-like receptors (TLRs) and inflammasome, as well as through different upstream kinase cascades via canonical or non-canonical pathways. IKKα/β and NIK are the most important upstream kinases, although several kinases can directly regulate the transcriptional capacity of NF-κB factors. IKKγ, generally called NEMO, is an important regulatory component of the IKK complex being linked upstream to genotoxic signals and IL-1 and TNF receptor mediated signaling. Activating kinases phosphorylate IκB proteins which are released from the complex and then degraded in proteasomes. Subsequently, the NF-κB complexes, having the crucial role of pleiotropic mediator of gene expression, translocate into the nucleus and transactivate the expression of special sets of target genes, codifying different SASP molecules, including pro-inflammatory cytokines, chemokines, adhesion molecules, eicosanoids, growth factors, metallo-proteinases, nitric oxide, etc. [25]. On the other hand, emerging experimental data principally performed on human skin fibroblasts have convincingly demonstrated the role of NF-κB signaling as the major pathway stimulating SASP phenotype [36-40]. Among the endogenous NF-κB inducers, a particular action is mediated by oxidative stress, DNA damage and immune defense, which are typical features of the entropic ageing process and age-related diseases [25].

**Figure 2:**
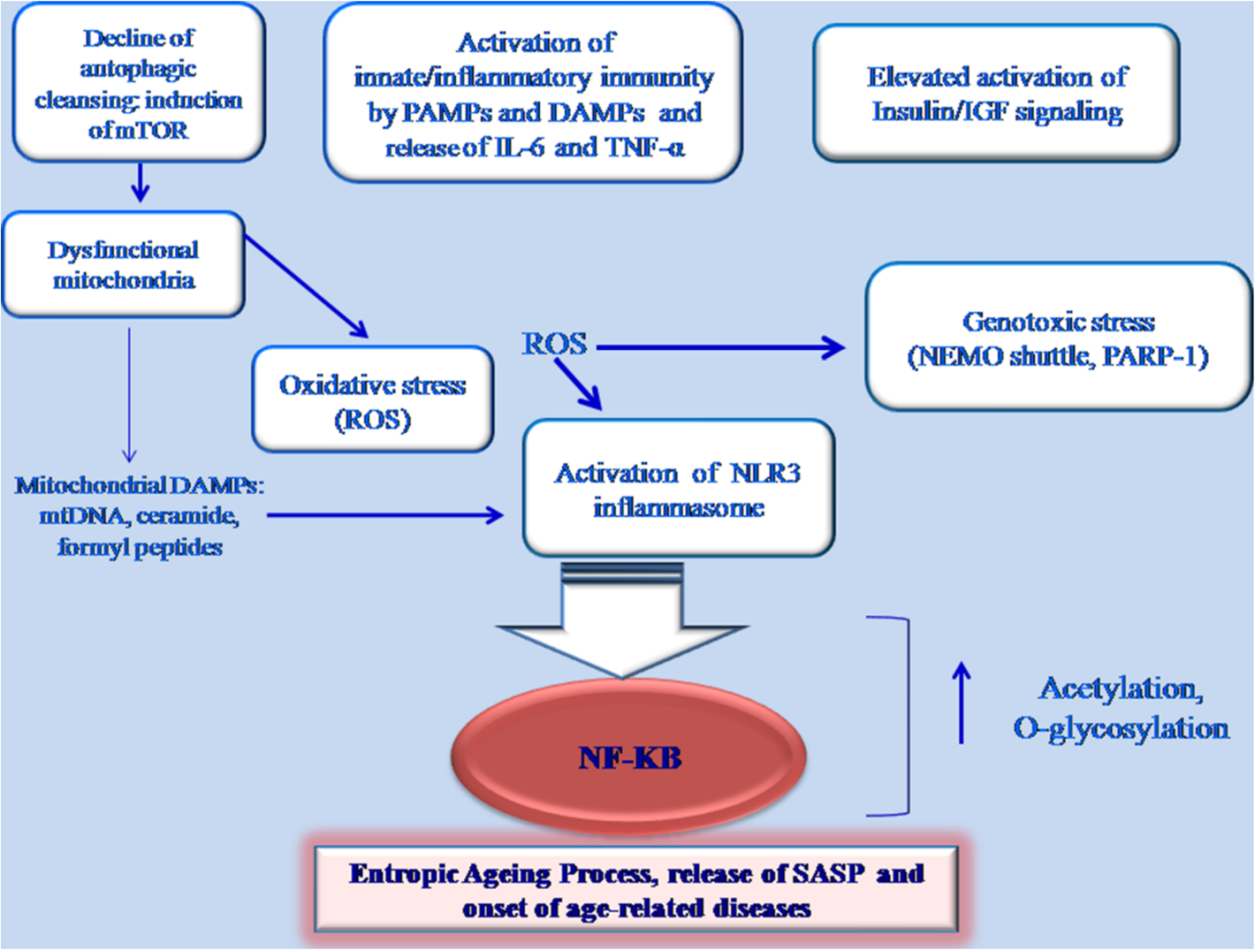
**NF-kB system is at the hub of aging inflammatory network Its activation is induced by different factors, such as mitochondria dysfunction, oxidative stress, activation of Inflammasomes, decline of autophagic cleansing.** Other NF-kB activators are activation of innate/inflammatory responses by PAMPs and DAMPs, elevated induction of insulin/IGF1 pathway, acetylation and O-glycosylation of components of NF-kB pathway, and DNA damage. NF-kB system induces entropic ageing process and release of SASP.

These observations lead in considering NF-κB as hub of ageing inflammatory network, whose the mentioned factors act as NF-κB activators and pro-ageing factors. With advancing age, these factors increase and determine a sustained NF-κB activation, eliciting a host defense “*catastrophe*”, responsible of SASP release. In turn, SASP, which occurs in several cells (i.e. fibroblasts, epithelial cells, endothelial cells, astrocytes, preadipocytes, and leukocytes as well as in postmitotic cells) participates, together the phenomenon of inflammageing, in the low chronic inflammation, improving both entropic ageing process and onset risk for age-related degenerative diseases, as result of harmful responses (i.e. chronic inflammatory responses, increased apoptotic resistance, decline in autophagic cleansing and tissue atrophy) (see Figure 2) [2,36,41]. Thus, chronic inflammation predisposes individuals to various age-related diseases. For example, the pro-inflammatory SASP of senescent endothelial cells has been proposed to contribute to CVD by initiating and fueling the development of atherosclerotic lesions. In addition, the expression of a SASP by astrocytes, which has been documented both in cells that were made senescent in culture as well as cells that were isolated from aged brain tissue, has been suggested to initiate or contribute to neuroinflammation, responsible of many neurodegenerative diseases, such as AD, causing or exacerbating age-related decline in both cognitive and motor function [42].

In the light of this evidence, the research has focused the attention on identifying pro-ageing factors, “*NF-*κ*B activators*”, as possible ageing biomarkers. Here, we describe some of them as promising inflammatory biomarkers of ageing and age-related diseases.

#### Oxidative stress, mitochondrial dysfunction, oxidative stress and activation of inflammasomes

Among the ageing modifications, mitochondrial alterations happen. They include an increased content of oxidation products and a diminished functional activity, conditions described and called as *mitochondrial dysfunction*[38,39]. An enhance of mitochondrial content in oxidation products, accompanies the entropic ageing process, and protein carbonyls, thiobarbituric acid reactive substances, ROOH and 8-hydroxy-2′-deoxyguanosine are the major markers. Recent experimental data on animal models, such as rats and mice, demonstrate the increase of these molecules with ageing in different tissues and organs [43]. In addition, current evidence underlines the increase of levels of several oxidative products in human biofluids, such as urine, serum, plasma, and blood, from old individuals than young subjects [44].

Mitochondrial dysfunction and oxidative stress are not associated only with ageing process, but also with the pathogenesis of several age-related diseases, as reported by recent experimental literature data [45-48]. Their detrimental effects are commonly attributed to disturbances in energy metabolism and increased Reactive oxygen species (ROS) production, and the crucial role of mitochondria in apoptotic cell death. In addition, mitochondria dysfunction and oxidative stress seem to provoke and potentiate inflammatory responses, even if the mechanisms remain elusive [38,39]. However, recent evidence sustains a crucial role of mitochondria in the regulation of innate immunity/inflammatory responses through different ways [49,50]. Among these, one is mediated by ROS which can induce the assembly of multi-protein inflammatory complexes called inflammasomes [49,50]. In particular, they activate Nod-like receptor protein 3 (NLRP3), a member of these complexes and a major sensor of cellular stress signals, such as ROS. Subsequently, NLRP3 triggers the caspase-1 mediated maturation of precursors of IL-1β and IL-18 cytokines [51,52]. Thus, an endogenous stress-related inflammation is activated, defined by Medzhitov as “*para-inflammation*” [53]. The exact mechanism involved in the ROS-induced NLRP3 activation is still unclear. It has been recently demonstrated by Zhou and colleagues that ROS could activate NLRP3 inflammasomes via the redox regulation of thioredoxin/thioredoxin-interacting protein balance [54]. In addition, the ROS can directly activate the inflammasomal pathways through the oxidation of thiol groups in leucine-rich repeat domain of NLRP3. Furthermore, under loss of mitochondrial integrity, mitochondria secrete DAMPs, such as ROS, ceramide, mitochondrial DNA and formyl peptides, which can also provoke activation of NLRP3 inflammasomes local and para-inflammation responses [55,56].

#### Decline of autophagic function and induction of mTOR

As described above, mitochondria with disrupted integrity and a deficiency in cellular housekeeping can activate through different ways NLRP3, and also NLRP1(another member of inflammasomes) in some tissues like brain, and stimulate inflammation [41,49,51]. In this context, the efficient function of autophagic uptake and lysosomal degradation of dysfunctional mitochondria should be a crucial element in maintaining tissue homeostasis [41]. Autophagy is, indeed, an ancient housekeeping mechanism, which regulates cellular homeostasis by facilitating the removal of misfolded proteins and dysfunctional organelles, such as mitochondria [57,58]. However, authophagic capacity seems to be compromised in ageing and age-related diseases, as proposed in “*garbage can*” hypothesis of Brunk and Terman [55]. On the other hand, there is growing evidence on inflammasome activation in many pathological conditions. Thus, a deficiency in authophagic housekeeping could trigger an inflammatory component and aggravate their pathogenesis [41,49,50,56-58]. After ten years of experimental work, the “garbage can” hypothesis still seems to be valid, since different research approaches have demonstrated clearly the decline of autophagy with ageing and the increased mitochondrial dysfunction[41,49,50,56-58]. Accordingly, the ageing decline in autophagy creates problems in cellular housekeeping functions, which stimulate NF-κB signaling directly or via inflammasomes trigger SASP and provoke the onset of entropic ageing phenotype [41]. Inflammatory NF-κB signaling seems also to have the capacity to repress autophagy and to induce this destructive interplay between autophagy and inflammasomes [41,49,50,56-58]. In particular, TNF-α can induce or repress autophagy in a NF-κB dependent manner. In presence of NF-κB signaling, TNF-α activates mammalian Target of Rapamycin (mTOR), a major autophagy inhibitor. On the contrary, in cells lacking of NF-κB activation, TNF-α stimulates the expression Beclin 1, an enhancer of autophagy [41,49,50,56-58].

TOR is a highly conserved serine/threonine kinase and a central controller of cell growth, metabolism and ageing. mTOR is activated in response to nutrients, growth factors and cellular energy. Deregulation of mTOR has been implicated in inflammation, ageing and several age-related diseases (i.e. cancer, metabolic syndrome, neurological diseases) [59]. It interacts with several proteins to form two distinct complexes named mTOR complex 1 (mTORC1) and 2 (mTORC2), differentially activated by distinct extracellular and intracellular signals. mTORC1 responds to amino acids, stress, oxygen, energy, and growth factors and is acutely sensitive to rapamycin. It promotes cell growth by inducing and inhibiting anabolic and catabolic processes, respectively, and also drives cell-cycle progression. mTORC2 responds to growth factors and regulates cell survival and metabolism, as well as the cytoskeleton. mTORC2 is insensitive to acute rapamycin treatment but chronic exposure to the drug can disrupt its structure. The activation of mTORC1 by growth factors and nutrients inhibits autophagy and promotes protein synthesis. Over time, this may promote cellular stress (protein aggregation, organelle dysfunction, and oxidative stress), which might lead to damage accumulation and a reduction in cell function and thus promote the development of aging-related diseases. Also, mTORC1 activation induces stem cell exhaustion, which reduces tissue repair and promotes tissue dysfunction [59].

#### Activation of innate/inflammatory response by PAMPs and DAMPs

During ageing, clonotypic immunity declines. This condition is defined *immune-senescence*[30]. In contrast, innate immunity seems to be efficiently activated and to induce a chronic inflammatory phenotype, as mentioned above [30]. The activation of innate immunity is mediated through the linking of pattern recognition receptors (PRRs), multi-ligand and evolutionarily conserved receptors (i.e. TLRs, NLRs and RIG-I-like receptors), with invading pathogen structures, called pathogen-associated molecular patterns (PAMPs), and endogenous danger molecules, the DAMPs [26]. This determines the release of different inflammatory mediators (i.e. IL-6 and TNF-α) by NF-κB pathway [25]. Among PRRs, TLRs, and mainly TLR4 and TLR2, recognize not only PAMPs, but also a large number of different alarmin age type DAMPs, including high mobility group box 1 (HMGB1), S100, heat shock protein (HSP)-60 and −70, and defensins [26,34]. In addition, both TLR2 and −4 have a key role in the pathogenesis of several age-related diseases [34]. Accordingly, variants of genes codifying these molecules seem to modify the susceptibility of age-related diseases and survival to extreme age, as recently described in our study [27]. On the other hand, the +896A/G (Asp299Gly; rs4986790) and +1196C/T (Thr399Ile; rs4986791) TLR4 SNPs have been phenotypically associated with changes in the production of pro- and anti-inflammatory cytokines, and principally the Asp299Gly SNP seems to have a key role in AD, prostate cancer, atherosclerosis and, reciprocally, in longevity [27,34].

Furthermore, an intriguing and innovative hypothesis has been recently suggested based on the crucial role of microRNAs in the dysfunction of TLRs signaling and the acquisition of SASP with NF-κB activation. Thus, these conditions can be considered as two interconnected phenomena [60].

During ageing, proteins, DNA and lipids, long-live macro-molecules can be targets of different age alterations. i.e. the Maillard reaction, a well known non-enzymatic glycosylation mechanism, induced as result of enhance of oxidative stress and hyperglycemia [2]. This results in the formation of protein glycation products, called AGEs (advanced glyaction end products), considered pro-ageing factors and activating NF-κB pathway by their linking with characteristic PRR receptors, the RAGE receptors (receptor for advanced glycation end products). With advancing age, AGE content increases in tissues. AGE process also improves in diabetes, atherosclerosis, neurodegeneration and several inflammatory diseases. The major harmful AGE effect in ageing seems to be the maintenance of anti-apoptotic and pro-inflammatory phenotype. Of special note is the glycation of collagen and elastin which seem to have a key role in vascular pathologies [26].

#### Induction of NF-κB signaling pathway by pro-inflammatory cytokines (mediated/or not by lipid rafts)

Activation of innate immunity in ageing process (see above) determines the production and release of SASP, such as different inflammatory molecules. Among these, pro-inflammatory cytokines are mostly observed to be at elevated level in elderly people. These cytokines can also activate the NF-κB pathway and this way can propagate and aggravate the inflammatory changes. IL-6 and TNF-α are clearly up-regulated with ageing, even if their exact role in the ageing process has been difficult to establish because of their complex and cell-type functions [25].

Recent evidence reported that NF-κB pathway activation via pro-inflammatory cytokines, and particularly via TNF-α, can be mediated by lipid rafts. Precisely, the binding of TNF-α to the TNF receptor (TNFR) results in receptor clustering within specialized domains at the cell surface, named as *lipid rafts*, which function as physical platforms for various molecules and are involved in a variety of biologic processes, such as molecular sorting, membrane trafficking, and signal transduction. For example, lipid rafts are important in the primary steps of T-cell antigen receptor signaling and B-cell antigen receptor signaling via triggering the phosphorylation of adaptor proteins [61]. The dynamic recruitment of ligand-bound receptors into lipid rafts has been suggested to be critical for the initiation of signaling transduction, including the NF-κB pathway. Legler and colleagues [62] reported that translocation of TNFR to lipid rafts is essential for TNF-α-mediated NF-κB activation, and disturbances in lipid raft organization switch the effect of TNF-α signaling from NF-κB activation to apoptosis, demonstrating that lipid rafts are crucial for the outcome of TNF-α-activated signaling pathways. In addition, lipid rafts have also been demonstrated to be required for NF-κB activation induced by IL-1, lipopolysaccharides, CD40L, or CD3/CD28, and the disruption of lipid rafts results in the inhibition of NF-κB activation mediated by these stimuli [63-65]. These studies indicate that lipid rafts play important roles in the activation of proximal NF-κB signaling.

#### Excessive stimulation of insulin/ insulin like growth factor (IGF) signaling

An excessive insulin/IGF signaling has been demonstrated to accompany ageing process [2]. Insulin/IGF signaling determines detrimental age effects via NF-κB pathway evoking activation of IκB kinase α/β complex. As consequence improving of inflammatory responses and resistance of apoptosis are induced. Given that impairing the signaling of insulin/IGF signaling pathway can activate the FOXO-dependent lifespan extension, this implies the role of NF-κB pathway in driving the ageing process via insulin/IGF axis [66].

#### Post-translation modifications of the members of NF-κB pathway

Members of NF-κB pathway are targets of several post-translation modifications. They influence both the activation of the pathway and transcriptional efficiency of NF-κB system [2,25]. Phosphorylation and ubiquitination are the major regulatory changes during activation. Acetylation, O-glycosylation and sumoylation can also control the transcriptional efficiency of NF-κB system during stress condition., i.e. inflammatory responses. In addition, increased protein acetylation can also activate cellular senescence***.*** On the other hand, molecules involved in cellular survival, such as the Sirtuin molecules (see below), and particularly SIRT 1 and 6, can deacetylate a NF-κB component, the p65, and repress NF-κB signaling [2,25].

Glucose tolerance decline, cause of insulin resistance and hyperglycemic disorders, determines O-glycosylation. Chronic hyperglycemia mediates glucotoxicity through AGE formation or via the production of O-linked N-acetylglucosamine (o-GlcNac)-modified proteins. On the other hand, levels of O-glycosylated proteins increase during ageing. In particular, an increased O- glycosylation of Ikkβ protein, able to enhance NF-κB activity, has been observed during ageing. O-glycosylation can also target p65 NF-κB protein and potentiate the transcriptional efficiency NF-κB components. This action is regulated by p53 protein, which can inhibit glycolysis and subsequently suppress the activation of Ikkβ/ NF-κB signaling [2,25].

#### DNA damages

One of the major stochastic age mechanism is genomic instability [2,67]. DNA lesions appear during ageing in both nuclear and mitochondrial DNA, as result of free radicals and oxidative stress. Under genotoxic stress, the major pathways activated are p53, NF-κB and PARP-1 (poly-(ADP-ribose)-polymerase-1) [67]. In particular, activation of NF-κB signaling represents one of the principal cellular features evoked by DNA damage [68]. The DNA damage-dependent NF-κB activation cascade is defined NEMO shuttle, since an essential NF-κB modulator (NEMO; as mentioned above) under genotoxic stress forms an complex with PIDD (p53-induced protein with a death domain) and RIP1 (receptor interacting protein) kinase [69]. This complex accumulates in nuclei and a nuclear matrix ligase (PIASy) can sumoylate the NEMO protein. Sumoylation is a prerequisite to allow a Ataxia telangiectasia mutated (ATM) kinase to phopshorylate NEMO protein. Subsequently, NEMO is desumoylated and the NEMO/ATM complex is exported from nuclei in cytoplasm where it activates Ikk kinases, by triggering NF-κB signaling. This consents to prevent the p53-induced apoptosis, since Ikk kinases phosphorylate p53 and induce its degradation by proteasomes [67-69].

Another hallmark of DNA damage is the induction of PARP-1 pathway, an ubiquitously expressed member of PARP family of enzymes able to modify proteins by poly(ADP-ribosyl)lation. PARP-1 is a sensor of DNA damage and maintains the genome integrity by regulating DNA repair [70]. In addition, PARP-1 is considered a novel co-activator of NF-κB signaling, which can potentiate the NF-κB activation in genotoxic stress [25,71]. Furthermore, it is one of the proteins involved in the regulation of the length of nucleoprotein structures located at the ends of chromosomes, the telomeres [71]. Telomeres are subject to shortening at each cycle of cell division and are highly sensitive to damage induced by oxidative stress. During ageing, both chronic inflammation and oxidative stress induce increased base oxidation. In contrast, to the majority of genomic DNA, there is evidence that telomeric DNA is deficient in the repair of single strand breaks. Thus, it creates a persistent damage of telomeres and a faster rate of telomere shortening, which induces cellular senescence and a faster rate of biological ageing. Since chronic oxidative stress plays a major role in the pathophysiology of several chronic inflammatory diseases, it has been hypothesized that telomere length is reducing at a faster rate during oxidative stress. On the other hand, telomere shortening has been assumed a biomarker of premature cell senescence in vascular and metabolic diseases [71,72]. Therefore, assessment of telomere length as well as the evaluation of both function and integrity of PARP-1 might be useful biomarkers of both biological ageing and disease onset and progression [71,72].

### Potential strategies against ageing and age-related diseases: drug and nutrition interventions and life-style modifications, and their effects on targets of inflammatory network

An excessive activation of NF-κB signaling pathway characterizes the entropic ageing process, responsible of inflamm-ageing and SASP phenotype, and the consequent onset of several age-related diseases [31-34,36]. This is plausible since nearly all insults enhancing the ageing process are well-known activators of NF-κB signaling system, as illustrated in Figure 2. The NF-κB signaling pathway also represents the lynchpin of host defense receiving the input signaling from the PRR receptors and subsequently organizing the transcriptional output response against the acute danger [25,26]. In both two cases, the sustained activation of NF-κB signaling pathway can trigger and enhance the entropic ageing process in many different ways, as above described [1,2]. Thus, the NF-κB system is at the hub of ageing process. This concept leads us in considering molecules and mechanisms linked to NF-κB signaling system as potential ageing biomarkers, as described. In addition, we also suggest them as targets for the development of new therapeutic strategies against ageing and age-related diseases.

On the basis of data reported herein, we proposed some suggestions on possible therapeutic drug and nutrition interventions and life-style modifications, and their effects on targets of inflammatory network (see Table 2).

**Table 2:** Potential therapeutic interventions and effects on targets of inflammatory network

**Therapies**	**Target effects**
**Monoclonal antibodies against these cytokines and their receptors**	Reduction of levels of IL-6, TNF-α
**Non-steroidal anti-inflammatory drug**	
**Agonists of cytokine receptors or PRR receptors for people who do not respond to (or comply with) NSAID therapy**	
**Antibody-mediated stimulation of the decoy TLR receptors, such as TAM receptors, or of the intracellular TLR regulators for people with pro-inflammatory alleles in TLR4 and TLR2 genes**	
**Statin therapy**	
**Physical activity**	
**Administration of prebiotics and probiotics**	
**Caloric restriction**	Decrease of oxidative stress
**Polyphenols**	
**Use of drugs having mimic CR action**	
**Caloric restriction**	Mitochondria biogenesis as preventive action against mitochondrial dysfunction
**Use of drugs having mimic CR action**	
**Polyphenols**	
**Caloric restriction**	Reduction of the activation of NF-KB pathway
**Terpenoids**	
**Resveratrol**	
**Use of specific miRNAs**	
**Administration of prebiotics and probiotics**	
**Use of drugs having mimic CR action Curcumin**	
**Caloric restriction: inhibition of mTOR pathway**	Preventive action on the possible reduced activity of autophagic cleansing
**Rapamycin: inhibition of mTOR pathway**	
**Curcumin: influences the mTOR pathway**	
**Caloric restriction**	Reduction of the excessive activation of Insulin/IGF1 pathway
**Metformin with CR mimic response**	

#### Anti-inflammatory drug interventions

##### ➢*Use of monoclonal antibodies and/or non-steroidal anti-inflammatory drugs*

The presence of “high-risk” levels of IL-6 and TNF-α in elderly people suggests the possibility to develop preventive measures using specific inhibitors, such as monoclonal antibodies against these cytokines and their receptors. Reduction of inflammatory mediators may be also induced through non-steroidal anti-inflammatory drug (NSAID) therapy. For people who do not respond to (or comply with) NSAID therapy, other more sophisticated preventive approaches may be possible, including the use of agonists of cytokine receptors or PRR receptors, i.e. TLR4 and −2, particularly in subjects carriers of high inflammatory responder alleles [27,34]. On the other hand, the activation of PRR receptors, such as TLR4 and −2, evocated by PAMPs or DAMPs particularly upon ageing, induce the release via NF-κB pathway of a large number of components of SASP, such as pro-inflammatory IL-6 and TNF-α cytokines [26,34]. In addition, the magnitude of cytokine production, and in general that of all pro-inflammatory mediators, has been shown to vary individually and is likely based on genetic heterogeneity. One or more functional SNPs in one or more innate immunty genes might be responsible. Accordingly, recent studies have suggested the role of +896A/G TLR4 SNP in cytokine production. In particular, high levels of pro-inflammatory cytokines were observed in carriers bearing the +896A/G TLR4 SNP [27,34].

Another possible therapeutic intervention in subjects with pro-inflammatory alleles of TLR4 and TLR2 genes might be antibody-mediated stimulation of the decoy TLR receptors, such as TAM receptors, or the intracellular TLR regulators (i.e. Supressor of cytokine signaling-SOCS molecules), involved in the inhibition of the inflammatory response, by mediating TLR degradation, or the activation of competitive or depho-sphorylation functions [73]. The sequential induction of these pathways, and their integration with upstream TLR and cytokine signaling networks, may limit the inflammatory response and maintain innate immune system homeostasis. A better understanding of the regulatory mechanisms of this cascade may have important implications for therapeutic intervention in human immune disorders and reduce the risk development for several age-related diseases [27,34].

##### ➢*Statin therapy*

Statin therapy has been demonstrated to have benefical effects in reducing primary and secondary CVD risk through the lipid-lowering, but also in inducing anti-ageing actions, such as inflammatory molecule lowering, especially IL-6 and CRP. On the other hand, results from Justification Trial Evaluating Rosuvastatin (JUPITER) confirmed that statin treatment in apparently healthy subjects with elevated CRP and non-elevated Low density lipoprotein cholesterol resulted in significant reduction in both these markers and CVD [74].

#### Nutrition interventions and life-style modifications

##### ➢*Caloric restriction*

Another possible anti-ageing strategy, able to reduce the biological effects of NF-κB signaling pathway in ageing, is the notable caloric restriction (CR) [75]. Restricting the intake of calories has been practiced as a method for increasing both the length and quality of life for over 500 years. Experimental work confirming the success of this approach in animals has accumulated over the last 100 years. CR may extend life by up to 50% in rodents, with progressively less impact the later in life it is started. This effect is matched by profound impacts on age-related diseases, including reduced risk of cancer, neurodegenerative disorders, autoimmune disease, CVD and T2DM [75]. The disposable soma theory of ageing suggests that CR evolved as a somatic protection response to enable animals to survive periods of food shortage [4]. The shutdown of reproductive function during CR is consistent with this suggestion, but other features of the phenomenon are less consistent with this theory. Some researchers have, indeed, proposed that in rodents it may be mostly an artifact of domestication. CR induces profound effects on animals at all levels from the transcriptome to whole animal physiology and behavior. Animals under CR lose weight which is disproportionately contributed to by white adipose tissue. Generally animals on CR change their activity patterns. Thus, they are more active prior to food delivery each day, but total activity may be unchanged or reduced [75]. Considerable debate has occurred over the effects of CR on resting metabolic rate (RMR). Total RMR declines, but as body mass and body composition also change it is unclear whether metabolism at the tissue level also declines, is unchanged or even increases. Body temperature universally decreases. Hunger is increased and does not seem to decline even with very long term restriction. Circulating adipokines are reduced reflecting the reduction in white adipose tissue mass under CR [75]. There is also a large reduction in circulating insulin and glucose levels. There are profound tissue level changes in metabolism with a generalized shift from carbohydrate to fat metabolism.

Four pathways have been implicated in mediating the CR effects. They are the insulin/IGF-1signaling pathway, the Sirtuin pathway, the adenosine monophosphate (AMP) activated protein kinase (AMPK) pathway and mTOR pathway [75]. These different pathways may interact and all play important roles mediating different aspects of CR response. Exactly how they generate the health benefits remains open for debate. However, one of the major impact of CR is the reduction of oxidative stress [76]. As described above, the major cellular source of ROS are the mitochondria. Isolated mitochondria from animals under CR show a reduced ROS production. In particular, CR results in an increase in the level and activation of adenine nucleotide translocase and uncoupling proteins able to reduce the mitochondrial membrane potential. This results in a decline in superoxide radical (O2) production and a less damage to the lipids in the mitochondrial membrane reduced ulteriorly by increases in the membrane lipid saturation [76]. Increases in superoxide dismutase convert superoxide into hydrogen peroxide and increased levels of Se-dependent glutathione peroxidase and catalase convert this to water reducing the production of the toxic hydroxyl radical (OH). Lowered levels of OH diminish the oxidative damage to proteins and DNA, which is further ameliorated by enhanced levels of degradation and base excision repair respectively [76]. Furthermore, CR induces mitochondrial biogenesis, as evidenced by changes in mtDNA levels, and protein levels [77]. Such effects on mitochondrial biogenesis are consistent with the idea that there may be a tissue level increase in oxygen consumption under CR which is accommodated in the reduced overall energy budget by the reduced amount of metabolizing tissue.

In addition, CR increases the levels of a member of Sirtuin family (SIRT1 to SIRT 7), NAD + dependent deactylases involved in the regulation of the activity of many proteins, energy metabolism, cell survival and longevity [78,79]. In particular, CR increases the expression of SIRT1 in multiple tissues, even if this effect does not appear to be uniform in all tissues or across different studies [80]. It has been demonstrated that SIRT1 interacts with p65/RelA protein and specifically cleaves the acetyl group form, the lysine-310 of p65 protein, involved in enhancing the trans-activation efficiency of NF-κB system [81,82]. Thus, SIRT1 is a potent inhibitor of NF-κB system.

An enhanced autophagy is also induced by CR via the inhibition of mTOR or the activation of AMPK pathway. This last is an evolutionary conserved sensor for disturbances in cellular energy balance and a major inductor of autophagy. Thus, CR acts directly or indirectly as inhibitor of NF-κB system [75,81,82].

Based on these observations, it is possible to assume that CR has beneficial effects, i.e. the extension of the average and maximum life span and delaying the onset of age-associated changes. However, this has been proven only in animal models, such as yeast, worms, flies and some mammals (rats and mice), and some criticisms (as above suggested) lead to consider it as an artifact of domestication, particularly in rodents [75,83-85]. In higher mammals, CR delays many diseases associated with aging including cancer, diabetes, atherosclerosis, CVD and neurodegenerative diseases [86,87]. The incidence of these diseases increases with age and they contribute significantly to mortality. Therefore, CR could increase life span by increasing the body’s general state of health and providing a nonspecific, resistance to chronic diseases and metabolic derangements [86,87]. However, the ultimate question, how does CR effect the human body, was studied in a limited number of experiments [88]. The study of CR effects on human longevity faces ethical and logistical challenges since the average life span is close to 80 years for the population in developed countries. Therefore, human studies are focused on measuring the CR-related changes that could slow the aging process and the progression of chronic diseases thus increasing life span. The most convincing evidence that CR could have a positive effect in humans was provided by experiments by Fontana and coworkers, by the Comprehensive Assessment of Long-Term Effects of Reducing Calorie Intake (CALERIE Phase 1), and by data obtained on the members of the Caloric Restriction Society [89-93].

Fontana and coworkers [89] assessed the effect of a 6-year long CR diet on risk factors for atherosclerosis in adult male and female adults (age range 35–82 years) and compared them to age-matched healthy individuals on typical American diets (control group). The total serum cholesterol level and low-density lipoprotein (LDL) cholesterol levels, the ratio of total cholesterol to high-density lipoprotein cholesterol (HDL), triglycerides, fasting glucose, fasting insulin, CRP, platelet-derived growth factor AB, and systolic and diastolic blood pressures were all markedly lower in the CR group. The HDL cholesterol was higher after CR. Medical records of individuals in the CR group indicated that, before they began CR, they had serum lipid-lipoprotein and blood pressure levels in the expected range for individuals on typical American diets, and similar to those of the comparison group. Thus, this study concluded that long-term CR can reduce the risk factors for atherosclerosis.

The effect of fat loss induced by either (a) a long-term 20% CR or (b) a 20% increased energy expenditure (IEE) by exercise on coronary heart disease (CHD) risk factors was detected in a one-year randomized, controlled trial on 48 non-obese male and female subjects. The CR or exercise induced reductions in body fat were quantitatively similar and were accompanied by similar reductions in most of the major CHD risk factors, including plasma LDL-cholesterol, total cholesterol/HDL ratio, and CRP concentrations. Thus, these data evidenced that long-term CR or IEE of the same magnitude lead to substantial and similar improvements in the major risk factors for CHD in normal-weight and overweight middle-aged adults [90].

The effects of a 1-year, 20% CR regime or 20% IEE by exercise, on the oxidative damage of DNA and RNA, was evaluated by white blood cell and urine analyses in normal-to-overweight adults. Both interventions significantly reduced oxidative damage to both DNA and RNA in white blood cells compared to baseline. However, urinary levels of DNA and RNA oxidation products did not differ from baseline values following either 1-year intervention program. The conclusion of the study was that either CR or IEE by exercise reduce systemic oxidative stress which is reflected in a decreased DNA or RNA oxidative damage [91].

CALERIE, a research program initiated by the National Institute on aging and involving three research centers, performed in the Phase 1 three pilot studies to determine whether long-term (6–12 months) effects of 20–25% CR in free-living, non-obese humans could be investigated and to evaluate the adaptive responses to CR. This randomized, controlled, clinical trial concluded that CR subjects had a lower body weight, a decreased whole body and visceral fat, a reduced activity energy expenditure, improved fasting insulin levels, improvements in cardiovascular disease markers (LDL, total cholesterol to HDL ratio, and CRP), and no change in bone density compared to controls [88]. In the ongoing CALERIE Phase 2, the researchers are testing whether 2 years sustained 25% CR of *ad libitum* energy intake results in beneficial effects, similar to those observed in animal studies [92].

Members of the Caloric Restriction Society (CRS) restrict food intake with the expectation that this would delay the disease processes responsible for secondary aging and to slow the primary aging process. Compared to age-matched individuals eating typical American diets, CRS members (average age 50 ± 10 yr) had a lower body mass index, a reduced body fat, significantly lower values for total serum cholesterol, LDL cholesterol, total cholesterol/LDL, and higher HDL cholesterol. Also fasting plasma insulin and glucose values were significantly lower than in the age-matched control group. Left ventricular diastolic function in CRS members was similar to that of about 16 years younger individuals. Chronic inflammation was reduced by CR and this was reflected in significantly lower levels of plasma CRP and TNF-α [88].

Aging is associated with a progressive reduction in heart-rate-variability (HRV)—a measure of declining autonomic function—and also a worse health outcome. The effect of a 30% CR on heart autonomic function was assessed by 24-hour monitoring of HRV in adults on self-imposed CR for 3 to 15 years and compared with an age-matched control eating a Western diet. The CR group had a significantly lower heart rate and significantly higher values for HRV. Also, HRV in the CR individuals was comparable to published norms for healthy individuals 20 years younger. The authors suggest that CR reset the balance between the sympathetic/parasympathetic modulation of heart frequency in favor of the parasympathetic drive thus increasing the circadian variability of heart rate [93].

Thus, in humans CR could delay many diseases associated with aging including cancer, diabetes, atherosclerosis, cardiovascular disease, and neurodegenerative diseases. As an alternative to CR, several CR mimetics have been tested on animals and humans, as described below*.*

##### ➢*CR mimetic drugs: biguanides, stilbenes and drugs*

Considerable effort has been directed in recent years to find drugs that mimic the CR response. Promising candidates are those that intersect with the critical signaling pathways identified above and include biguanides such as metformin, capable to target insulin signaling pathway, stilbenes (e.g. resveratrol) affecting sirtuin activity and drugs such as rapamycin that interact with mTOR signaling. Whether it will ever be possible to find drugs that capture the health benefits of CR without the negative side-effects remains unclear. Moreover, even if such drugs are developed how the current licensing system for drug use in western societies would cope with them may be a further obstacle to their use [75,88,94-96].

##### ➢*Polyphenols and resveratrol (a stilbene phytochemical)*

As mentioned above, several plant derived, folk medical compounds and extracts have been claimed to have anti-ageing effects [75,88,97-101]. However, only a small number of traditional remedies has been subjected to a clinical trial. Recently, many promising compounds have been identified and scrutinized. Among these, there are polyphenols (i.e. flavonoids and terpenoids), the major ingredients of fruits, vegetables and different spices [75,88,97-101]. Many of polyphenols are inhibitors of NF-κB signaling system, since they are potent antioxidants, and as consequence they can inhibit ROS production and activation of NF-κB signaling system [97-101]. Some of them (i.e. terpenoids) can also directly inhibit Ikk/ NF-κB signaling [97-101]. Accordingly, it has been found that low-doses of terpenoids can trigger cellular stress response and subsequently induce adaptive stress resistance, condition defined hormesis [97-101]. Stress resistance involves several molecular adaptations via the activation of AMPK pathway and the subsequent increase in the expression of survival genes, such as Sirtuins, FOXOs and p53 [97-101]. Of special note is the effect of resveratrol, a stilbene phytochemical. It induces activation of SIRT1 via AMPK pathway, and indirectly inhibition of NF-κB signaling system via the activation of survival genes [97-101].

##### ➢*Curcumin*

It has been postulated that a natural agent, curcumin, could influence cellular senescence [102]. Curcumin has attracted the attention of researchers and clinicians as an anti-inflammatory and anti-oxidant agent with a potential use in the therapy of many diseases with an inflammation constituent, e.g. cancer, CVD, AD, rheumatoid arthritis and metabolic syndrome. A plethora of studies using animal and cell line models have been undertaken to elucidate the molecular mechanisms and biological effects of curcumin and some clinical trials are underway. Sikora and colleagues [103] proposed that curcumin might act as an anti-aging agent not only by inhibition of NF-κB, but also by indirect influence on cellular senescence via mTOR. However, they also showed that conversely curcumin can induce senescence in colon cancer cells [104]. Moreover recent studies by Quitschke [105] also revealed the prosenescence activity of curcumin. Nonetheless, curcumin has many molecular targets and evokes a biphasic hormetic dose–response [106]. Thus, one cannot exclude that much lower concentration of curcumin than that used in these studies will inhibit/postpone cellular senescence or, at least, through NF-kB inhibition will reduce SASP. Interestingly, curcumin was shown to prolong life of model organisms such as Caenorhabditis elegans and Drosophila melanogaster, but did not influence (similarly to resveratrol) the life span of mice [107].

##### ➢*Physical activity*

Promising evidence suggests a role of physical activity in reducing the levels of inflammatory markers. Several speculations have been advanced[108-111]. However, the mechanisms underlying its anti-inflammatory effects seem complex and not fully elucidated. It has been recently considered that the decreased production of proinflammatory cytokines may originate from a reduction of adiposity, or the release of muscle derived IL-6 [108-111]. This last seems to induce several metabolic adaptations, i.e. hepatic glycogenolysis and lipolysis, and the release of cytokine inhibitors (i.e. IL-1ra, sTNFR and IL-10) and cytokine with potent anabolic effect, as IL-15 [108-111].

##### ➢*Probiotics and/or prebiotics*

Administration of probiotics and/or prebiotics to elderly seems to induce changes in several inflammatory parameters (i.e. pro-inflammatory cytokine lowering, CRP reduction), demonstrating that manipulation of gut microbiota may result in modification of functionality of an aged immune system. On the other hand, intestinal microbiota seems to play a fundamental role in maintaining human health. Its supposed importance in human physiology has recently led to label human subjects as “metaorganisms” because of their close symbiotic relation with indigenous gut microbiota. The “metaorganisms” hypothesis evidences the use of dietary supplementation with probiotics and prebiotics, as therapeutic strategy to preserve human health particularly during that life period not foreseen by evolution- “ageing”-, that inexorably alters gut microbiota composition, stability and functionality [112,113].

## Conclusions

From all observations described above, chronic inflammation has been proposed as the major biological mechanism underpinning the entropic ageing process and age-related diseases [31-34,36]. Inflammation is also the key response of host defense against pathogens and tissue injury [25,26]. In addition, it is current opinion that during evolution the host defense and ageing process have become linked together [2]. Thus, the large array of defense factors and mechanisms linked to the NF-κB system seem to be involved in the entropic ageing process [2,25]. This concept leads us in proposing inductors of NF-κB signaling system as potential ageing biomarkers and promising targets for the development of new therapeutic strategies against ageing and age-related diseases. In this report, we describe some inflammatory mechanisms linked to NF-κB signaling system as potential ageing biomarkers. In addition, some suggestions on their role as promising targets for the development of new therapeutic strategies have been discussed. Our interest has been, particularly, focused on possible interventions on molecular survival and resistance stress pathways, capable to reduce or inhibit NF-κB signaling pathway. However, it is not impossible to predict, whether these possible interventions (appropriate and specific drug therapies, lifestyle modifications, use of CR mimetics and other preventive therapeutic strategies) can very reduce or retard the onset of ageing biological phenotype and the onset risk of age-related diseases. Different motivations lead us to have prudence. Firstly, the major literature data on anti-ageing effects of therapeutic strategies have been obtained from studies on animals. Thus, potential therapy interventions on the basis of pathways identified in model organisms may be an illusion, because gains in longevity achieved in these organisms seem to decline with organismal complexity or depend on idiosyncratic physiology. Furthermore, lifespan in some organisms may be less plastic than in others. In addition, there are still enormous gaps in our knowledge about how metabolic pathways operate and interact. Serious side effects may constrain the effectiveness of pharmacological interventions.

The best treatment might be that which consents the repair of macromolecular damage. However, it is not clear that all toxic lesions associated with ageing process have been identified, or whether practical and appropriated strategies exist to eliminate them, as those mentioned above. Today, the researchers are becoming to speculate the concept based on reprogramming cellular senescence as way of organism rejuvenation or at least to alleviate age-related diseases considering cellular senescence as target model [114-116]. This hope derives by results of recent studies on progeroid mice demonstrating the possibility to reverse progeroid phenotype through genetic manipulation. This intervention of avoiding or reversing cellular senescence is based on induced pluripotent stem cell technology, which opened a new avenue of autologous regenerative medicine and the possibility to activate telomerase and change the telomere length [117,118]. Accordingly, other studies are needed to confirm and extend these current data. For example, genomic, transcriptomic and epigenetic investigations may eventually lead to better understanding the molecular and cellular inflammatory mechanisms associated with biological ageing. In addition, for the development of anti-ageing therapies for human, it should be more appropriate identifying cellular and serum ageing biomarkers and potential targets using apposite study model, such as centenarian offspring, healthy elderly people with a family history of longevity, as recently suggested [119]. On the other hand, the research of biomarkers of ageing and age-related diseases in understanding the health trajectories of the oldest-old is unexplored territory. It is important that this lacuna is filled given the rapid growth in the number of very old people in many contemporary populations. The goal of this research might guarantee improving of life quality rather than searching the elixir of long life.

## Competing interests

The authors declare that they have no competing interests.

## Authors’ contributions

CRB wrote the drafts of this manuscript and revised the intellectual content; GC contributed to collection of literature data. CRB had the overall supervision of the review processing. All authors edited the paper and approved its final version.
